# Validity of the Dictionary of Occupational Titles for Assessing Upper Extremity Work Demands

**DOI:** 10.1371/journal.pone.0015158

**Published:** 2010-12-03

**Authors:** Lonneke Opsteegh, Remko Soer, Heleen A. Reinders-Messelink, Michiel F. Reneman, Corry K. van der Sluis

**Affiliations:** 1 Department of Rehabilitation Medicine, Center for Rehabilitation, University Medical Center Groningen, University of Groningen, Groningen, The Netherlands; 2 Center for Rehabilitation ‘Revalidatie Friesland’, Beetsterzwaag, The Netherlands; Tulane University, United States of America

## Abstract

**Objectives:**

The Dictionary of Occupational Titles (DOT) is used in vocational rehabilitation to guide decisions about the ability of a person with activity limitations to perform activities at work. The DOT has categorized physical work demands in five categories. The validity of this categorization is unknown. Aim of this study was to investigate whether the DOT could be used validly to guide decisions for patients with injuries to the upper extremities. Four hypotheses were tested.

**Methods:**

A database including 701 healthy workers was used. All subjects filled out the Dutch Musculoskeletal Questionnaire, from which an Upper Extremity Work Demands score (UEWD) was derived. First, relation between the DOT-categories and UEWD-score was analysed using Spearman correlations. Second, variance of the UEWD-score in occupational groups was tested by visually inspecting boxplots and assessing kurtosis of the distribution. Third, it was investigated whether occupations classified in one DOT-category, could significantly differ on UEWD-scores. Fourth, it was investigated whether occupations in different DOT-categories could have similar UEWD-scores using Mann Whitney U-tests (MWU).

**Results:**

Relation between the DOT-categories and the UEWD-score was weak (r_sp_ = 0.40; p<.01). Overlap between categories was found. Kurtosis exceeded ±1.0 in 3 occupational groups, indicating large variance. UEWD-scores were significantly different within one DOT-category (MWU = 1.500; p<.001). UEWD scores between DOT-categories were not significantly different (MWU = 203.000; p = .49).

**Conclusion:**

All four hypotheses could not be rejected. The DOT appears to be invalid for assessing upper extremity work demands.

## Introduction

Hand injuries may severely influence a person's work capacity, frequently resulting in long periods of absence from work [1]–[4]. Functionality of the hands is crucial in most jobs. However, the type of functionality most needed may differ between and even within jobs. For example, grip force and gross movement coordination may be essential for construction workers, while fingertip dexterity and sensitivity may be more important in mechanics or surgeons. Hand performance in subjects with simulated finger disabilities has been investigated previously and it was found that strength was not influenced by the fictitious injury. Dexterity, lifting and some torque exertion tasks (e.g.: screwdriver handling) were negatively influenced by the diminished hand function [5]. The type of control to be handled during work might also determine whether the worker can perform the tasks at the required pace for the job [6]. Both aforementioned studies suggest that strength is not the only key factor determining whether a person can resume his job after a hand injury, but that other factors may be equally or even more important.

The Dictionary of Occupational Titles (DOT) [7] is frequently used in vocational rehabilitation and disability evaluations. It assists in the determination of the type and level of work a worker can perform, given his/her disability and employment history. The DOT can be used to find an occupation with demand levels that match the workers' functional abilities [8]. Based on the physical demands, the DOT classifies jobs into five categories: sedentary, light, medium, heavy and very heavy [8], [9]. These categories are suggested to be mutually exclusive. The physical demand ranking is mainly based on intensity, force, duration of material handling and energy expenditure. This ranking implies that within one DOT-category physical demands are similar and that workers practicing the same occupation have similar physical work demands. However, it is questionable whether these assumptions are applicable in patients with hand injuries. For example, some physical therapists practice massage therapy and may therefore have high hand work demands, while others use a ‘hands off ‘approach and have substantially lower hand work demands. Subjects with fictitious hand injuries [5], [6] would probably meet the requirements put forth by the DOT, since strength was not influenced by the injury, although they would probably be unable to perform many tasks. As such, the validity of the DOT in hand injured patients should be explored.

In 1980, validity of the DOT was already challenged: the classification seemed to be designed for unskilled factory and physical laboring jobs. It was stated that the DOT-classification did not appear to capture the full range of variability in the working conditions and physical demands of jobs [8]. For instance, while the ability to use hand function greatly determines whether a person can perform his/her job adequately, hand function is not taken into account in the descriptions of occupations in the DOT and in ranking the physical demands.

When a hand-injured patient needs to be advised concerning possible job changes using the physical demands ranking of the DOT, it should first be confirmed whether the DOT-categories are valid representations of upper extremity work demands. The physical demands ranking of the DOT implies that within each category workers have similar physical work demands. Furthermore, the DOT implies that workers in different DOT-categories have distinct work demands, but upper extremity work demands are not taken into account in this classification. It should also be confirmed whether a classification based on occupation is adequate, as workers can perform jobs differently, with different upper extremity work demands.

The aim of the current study was to determine whether the DOT is valid for assessing upper extremity work demands. Four hypotheses were formulated and tested in this study. If these hypotheses could not be rejected, then this would be interpreted as invalidity of the DOT for assessing upper extremity work demands.

1. Relationship between the DOT-categories and upper extremity work demands is weak;
2. Large variance in upper extremity work demands is possible within occupational groups;
3. Within DOT-categories substantial differences in upper extremity work demands can be found;
4. Between DOT-categories jobs can have similar upper extremity work demands.

## Methods

A database including 701 healthy workers was used [9]. Subjects were 20 to 60 years of age and were working in a wide range of professions. Subjects were recruited via local press and personal networks between 2005 and 2008. DOT-codes assigned to occupations of subjects were included in the database. Sociodemographic and occupational information were collected and all subjects completed the Dutch Musculoskeletal Questionnaire (DMQ) [10], [11].

### Ethics Statement

This study was approved by the Medical Ethical Committee of the University Medical Center Groningen, The Netherlands. All participants gave written consent; data was coded and analyzed anonymously.

### Materials

The DMQ is a questionnaire for the analysis of musculoskeletal workload and associated potential hazardous working conditions as well as musculoskeletal symptoms in worker populations. One of the domains taken into account is musculoskeletal workload, expressed in questions about postures, forces and movements [11]. Convergent and divergent validity were fair in the original questionnaire, evidence for concurrent validity was found [11] and the questionnaire appeared able to identify levels of exposure for specific movements [10], [11]. All upper extremity-related items from the shortened version of the DMQ [10], [11] were used to develop an Upper Extremity Work Demands (UEWD) score. The UEWD-score had to be developed, as no suitable instrument was available to measure upper extremity work demands. The upper extremity-related items from the shortened version of the Dutch Musculoskeletal Questionnaire (DMQ) were selected and summed. To select the set of items, three authors (LO, CS and RS) independently scored whether an item: 1) involved the upper extremities, and 2) whether the item measured a type of physical work demands (i.e.: duration, repetition, awkward positions, forceful movements and static muscle contractions). Comparison of these independent ratings revealed that complete agreement was reached. After calculating inter-item correlations between the selected items, the item lifting/pulling/pushing/carrying very heavy loads (>25 kg) was excluded as it correlated highly (>.80) with lifting/pulling/pushing/carrying heavy loads (>5 kg). The seven remaining items were summed, thereby creating an UEWD total score ranging from 7 (lowest upper extremity work demands) to 28 (highest upper extremity work demands). An English translation of the selected items is given in Appendix S1.

### Analyses

All statistical analyses were performed with Statistical Package for the Social Sciences (SPSS) 16.0. Data distribution was checked for normality (Kolmogorov-Smirnov Test: p≥.05).

#### Hypothesis 1

Spearman correlations were calculated to test the relation between the DOT and upper extremity demands as expressed by the UEWD scores. If the Spearman correlation coefficient was less than 0.50, the correlation was interpreted as weak and the hypothesis was not rejected.

Subsequently, three steps were taken to select the occupations in order to test the second, third and fourth hypotheses. 1) Cases were excluded if occupational information was missing. 2) Only occupational groups consisting of at least 10 subjects were included to make comparisons possible. 3) Median UEWD scores of the occupational groups were ranked (Table 1).

**Table 1:** Upper Extremity Work Demands-scores of occupational groups.

Occupation (n)	UEWD Median (IQR)	Kurtosis (SE)	z-score Kurtosis	DOT
Driving Instructors (n = 14)	8.0 (7.0 to 9.0)	6.00 (1.154)	2.28	2
Teacher (n = 34)	11.0 (8.0 to 13.0)	−.81 (.788)	−1.02	2
Job Consultant (n = 10)	12.0 (8.0 to 15.3)	−1.39 (1.334)	−1.02	2
Secretary (n = 21)	12.0 (11.0 to 14.5)	.42 (.972)	.67	1
Physical Therapist (n = 22)	13.0 (11.0 to 15.5)	−.63 (.953)	−.81	3
Nurse (n = 16)	13.5 (9.3 to 16.0)	−.34 (1.091)	−.56	3
Analysts (n = 11)	16.0 (14.0 to 19.0)	−.51 (1.279)	−.63	2
Home Attendant (n = 17)	18.0 (12.5 to 19.5)	−.59 (1.063)	−.74	3
Production Workers (n = 24)	17.5 (16.0 to 19.8)	.64 (.918)	.84	3
Surgery Assistant (n = 16)	20.0 (16.3 to 22.8)	−.59 (1.091)	−.74	2

*Note* UEWD Upper Extremity Work Demands score derived from Dutch Musculoskeletal Questionnaire; SE Standard Error; range of the UEWD score: 7 to 28; IQR Inter Quartile Range; Table sorted on the UEWD-score.

#### Hypothesis 2

Variances in UEWD-scores of occupational groups were visually inspected to test this hypothesis with the use of boxplots, and z-scores of kurtosis were calculated [12]. When kurtosis exceeded ±1.0, this was considered as large variance and the hypothesis was not rejected.

#### Hypotheses 3

For the within DOT-category comparison, two occupational groups from the same DOT-category that showed the largest difference in mean UEWD scores were selected. If data met the criteria for normality, independent t-tests were performed. If absence of normality was found, Mann Whitney U tests were performed.

#### Hypotheses 4

For the between DOT-category comparison, two occupational groups from different DOT-categories with the smallest difference in mean UEWD score were selected. If data met the criteria for normality, independent t-tests were performed; otherwise Mann Whitney U tests were performed.

## Results

Twenty-five subjects were excluded from the database because the UEWD-score could not be calculated, leaving 677 subjects to include in the analyses. UEWD scores were not normally distributed. 117 subjects were classified in DOT 1 (sedentary), 228 subjects were classified in DOT 2 (light), 284 subjects were classified in DOT 3 (medium) and 48 subjects were classified in DOT 4 (heavy). None of the subjects was classified in DOT 5 (very heavy). Mean age of the subjects was 41.3 years (SD ±10.4 years) and 433 subjects were men (64%).

### Hypothesis 1

**Figure 1:**
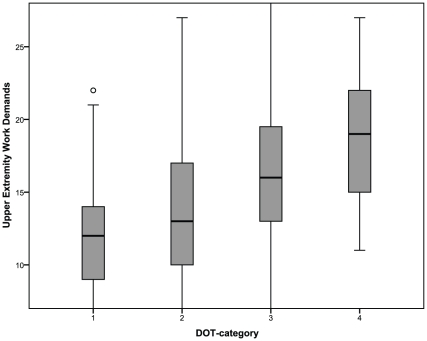
Upper Extremity Work Demands-scores per DOT-category. DOT Dictionary of Occupational Titles; Scoring range of the Upper Extremity Work Demands: 7–28.

Spearman correlation coefficient between DOT-categories and UEWD-scores was r_sp_ = 0.40 (p<.001). Boxplots are displayed in Figure 1. Based on these results, only a weak association could be found between the DOT-categories and UEWD-scores; therefore, hypothesis 1 was not rejected.

Next, the three steps, as described in the methods, resulted in a selection of 10 occupational groups in DOT-categories 1 to 3. A total of 210 subjects were excluded because job information was missing. Subsequently, occupational groups consisting of less than 10 subjects were excluded, thereby excluding 282 subjects. UEWD-scores of the remaining 185 subjects ranged from 7 to 28 (Table 1).

### Hypothesis 2

**Figure 2:**
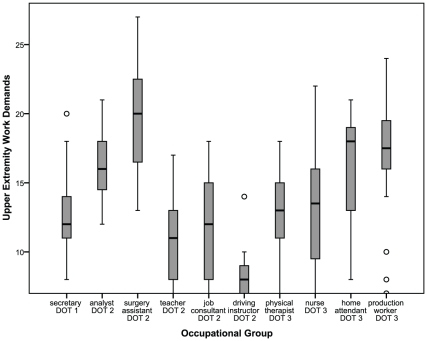
Variance in occupational groups. DOT Dictionary of Occupational Titles; Scoring range of the Upper Extremity Work Demands: 7–28.

Boxplots of UEWD-scores of all occupational groups are displayed in Figure 2. These boxplots show that large variances do occur in most occupational groups. Z-scores of kurtosis was calculated, thereby making comparisons between the occupational groups possible [12]. Z-scores of kurtosis exceeded ±1.0 in teachers, job consultants and driving instructors, indicating a non-normal distribution. Z-scores of the teachers and job consultants were negative, indicating a flat distribution, meaning that large variance was found; z-score of driving instructors was positive, indicating a pointy distribution, meaning that small variance was found (Table 1). In conclusion, large variances in UEWD-scores are possible in occupations, and therefore hypothesis 2 was not rejected.

### Hypothesis 3

**Figure 3:**
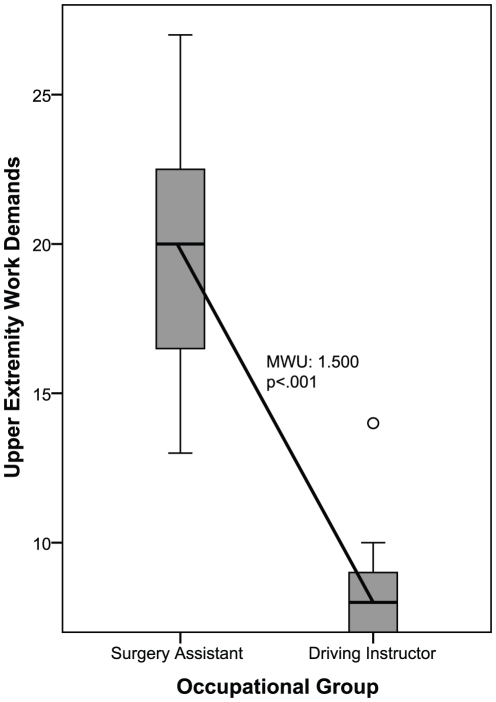
Upper Extremity Work Demands-scores within a DOT-category. MWU Mann Whitney U test; Scoring range of the Upper Extremity Work Demands: 7–28.

To compare occupational groups within one DOT-category, driving instructors and surgery assistants were selected, because they are both classified in DOT 2, and they have the largest difference in UEWD-scores. Mann Whitney U test demonstrated that differences in UEWD-scores between driving instructors and surgery assistants were significant (MWU  = 1.500; p<.001) (Figure 3). Based on these results, it was concluded that within one DOT-category differences in UEWD-scores can exist; therefore, the third hypothesis is not rejected.

### Hypothesis 4

**Figure 4:**
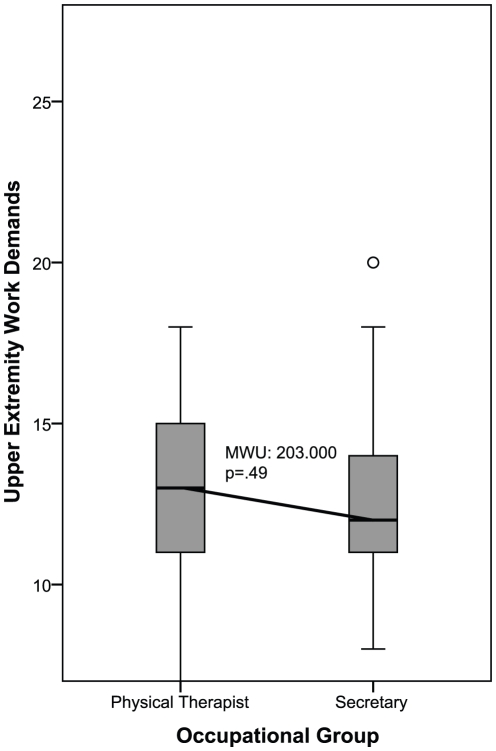
Upper Extremity Work Demands-scores between two DOT-categories. MWU Mann Whitney U test; Scoring range of the Upper Extremity Work Demands: 7–28.

To compare UEWD-scores of occupational groups between two DOT-categories, secretaries and physical therapists were selected. Even though secretaries are classified in DOT 1 and physical therapists in DOT 3, their mean UEWD-scores were similar. No significant difference in UEWD-scores between secretaries and physical therapists (MWU  = 203.000; p = .49) was found (Figure 4). These results indicate that occupations in different DOT-categories can have similar UEWD-scores; therefore hypothesis 4 is not rejected.

## Discussion

In this study the validity of the physical demands classification of the DOT for assessing upper extremity work demands was questioned. After testing four hypotheses in a large database of healthy workers, we concluded that the DOT is invalid for assessing upper extremity work demands, since none of the hypotheses could be rejected.

Each occupation in the DOT is identified by a nine-digit code, representing a classification structure based on the type of work performed (first three digits) and the complexity of work in relation to data, people and things (the second three digits); the final three digits are a unique numerical identification for each occupation, including physical work demands [8]. Physical demands can be divided in strength (lifting, carrying, pulling and pushing), climbing (climbing and balancing), stooping (stooping, kneeling, crouching and crawling), reaching (reaching, handling, fingering and feeling), talking (talking and hearing) and seeing (seeing) [8]. Yet, despite this wealth of information, the classification of physical work demands is only based on the strength, thereby possibly over-simplifying physical work demands. The DOT classifies physical work demands into 5 categories, based on the strength component, not taking any other components into account. For patients with hand injuries, this classification may not be valid, as they often have difficulties with handling objects, which is classed under the reaching component.

Four hypotheses were tested to investigate validity of the physical demands classification of the DOT.

### Hypothesis 1

Although an association was found between the DOT-categories and UEWD, the association was weak and the variance in each DOT-category appeared to be large. According to the DOT, categories are composed in such a way that they are exclusive in physical work demands, and have a strict lower limit. However, UEWD-scores overlap between DOT-categories and in category 1 to 3 the minimum score of 7 occurred. Therefore, it is not possible to give a vocational advice to patients with hand injuries, based on the DOT classification. Only hand injured patients who are working in a DOT-4 classified job may be advised about work ability using the DOT, as an UEWD score below 11 did not occur in category 4. If a person does not have the capacity to fulfill these work demands, he/she will need to switch to a less demanding job, that is category 1 to 3 or adjustments should be made to reduce the upper extremity work load.

### Hypothesis 2

In driving instructors a high kurtosis was found, indicating little variability in upper extremity work demands when performing the job. Other occupational groups had flat distributions (negative kurtosis), indicating heterogeneity in UEWD. The findings demonstrate that large variances are possible, although this is not present in all occupational groups. In some occupations many ways of accurately performing tasks exist, while in other occupations options are limited.

### Hypothesis 3

Surgery assistants and driving instructors, who were both classified in DOT 2, significantly differed on UEWD. These results indicate that upper extremity work demands can not be assessed with the DOT accurately. It appears reasonable that surgery assistants and driving instructors report different UEWD-scores, and therefore it would also be more logical if they would be categorized in different categories when considering upper extremity work demands.

### Hypothesis 4

Secretaries and physical therapists, which were classified in DOT 1 and DOT 3, had similar UEWD-scores. This is remarkable, as the DOT states that the physical demands categories are mutually exclusive. Based on our UEWD-scores it was expected that these occupations were classified in the same DOT-category.

Our findings demonstrate that the physical demands classification system of the DOT is not valid for assessing upper extremity work demands, and thus, can not be used validly to advice patients with hand-injuries or other complaints of the upper extremities on work ability. A specification of the physical demands classification may be needed, taking a full range of upper extremity work demands into account. The necessary information is, at least partly, available in the DOT, but not taken into account.

A limitation of our study is the absence of published evidence for the validity of the UEWD-scoring, as this has not been investigated. We constructed this scoring based on the DMQ, which has been validated [10], [11]. Items were selected based on content and adapted from a dichotomous to a 4-point Likert scale to gain more insight in the extent the workload appeared to be present in the workers job. In the original DMQ questionnaire a sum score is calculated; in the current study a sum score was calculated from a subset of items relating to upper extremity use. It is unknown how selecting questions from a questionnaire and re-calculating a sum score effects validity. However, it has previously been found that assessing physical exposure in the upper extremity using a self-report questionnaire is moderately reliable [13].

A second limitation of our study is the fact that no DOT 5 (Very Heavy) workers were present in the database, probably because these occupations are not performed often in the Netherlands. To be classified in DOT 5, a worker should lift or carry weights of 45 kg occasionally, and/or lift or carry 23 kg frequently, and/or lift or carry 9 kg constantly [7]. Most companies in The Netherlands comply with the Dutch laws on worker safety, based on the lifting guidelines of the National Institute for Occupational Safety and Health, in which a maximum lifting weight of 23 kg is advised [14]. Final limitation is that sample sizes of the four DOT-categories were unequal. Categories 1 to 3 were of adequate size to perform analyses, but DOT 4 was of insufficient size, as only 48 subjects were included and no occupational groups of at least 10 workers were present. As such, there could be a selection bias in our database, leading to more subjects with sedentary to medium occupations. Occupations identified in our DOT 4 group were, for example, farm worker (DOT-code: 410.684-010), tree planter (forestry) (DOT-code: 452.687-018), slaughterer/butcher (DOT-code: 525.381-014), baker (DOT-code: 526.381-010), structural-steel worker (DOT-code: 801.361-014) or stage technician (DOT-code: 962.261-014).

### Clinical Message

From our results it became clear that the DOT can not be used validly to guide vocational decisions for people who are limited in functioning due to hand and upper extremity problems. A classification based on occupation may not be feasible in all occupational groups when assessing upper extremity work demands, realizing that there are different strategies to perform activities. Individual cases or workplaces at one company should be assessed, although the psychometric properties of such assessments are questionable or absent. It can be assumed that similar job functions within one company are more closely related than functions between different companies. Therefore, we suggest a tailored classification for each company. A tool should be developed facilitating construction of such adapted classifications. An alternative could be a classification based on tasks, but even then workers may have different methods to perform the task. So far, the best and most reliable method to assess upper extremity work demands would be the workplace assessment, even though expensive and time consuming [15].

Besides solely work demands, the capacity of the person should be investigated if valid statements concerning the matching of worker and job are to be made. Functional capacity evaluations (FCE) are suitable instruments to evaluate work capacity. However, FCE's assess general physical capacity. It is unknown whether general physical work demands are related to upper extremity demands, and whether capacity of the upper extremities is somehow related to general functional capacity, as measured by a FCE [16]. These relations should be investigated in future studies. Earlier, it has been suggested to develop job-specific FCE-test protocols [17]. We suggest combining such a job-specific protocol with a body region-specific FCE-test protocol, such as for the hands. Some subtests of the FCE are specifically aimed at evaluating hand function [16]. Suitable advice can be provided only when both the demands and capacity for work are known. For as long as no suitable classification is available, workplace assessments should be administered to evaluate upper extremity work demands and combined with FCE's when advising patients with problems to the upper extremities. As workplace assessments are expensive and time-consuming, future studies should investigate more efficient and cost-saving clinical procedures to assess work demands.

## Supporting Information

Appendix S1 **Selected items to construct the Upper Extremity Work Demands-score.**
(DOC)
