# Supplementary material for: Validity of the Dictionary of Occupational Titles for Assessing Upper Extremity Work Demands
Source: PLoS One. 2010 Dec 3;5(12):e15158. doi: 10.1371/journal.pone.0015158 (PMC2997074; doi:10.1371/journal.pone.0015158)
Supplement: Appendix S1 — Selected items to construct the Upper Extremity Work Demands-score. (DOC) [file pone.0015158.s001.doc]

**Appendix S1.** Selected items to construct the Upper Extremity Work Demands-score.

|  |  | *Rarely or never* | *Sometimes* | *Often* | *Almost always* |
| --- | --- | --- | --- | --- | --- |
|  | **During work, do you have to:** |  | | | |
| 1 | Lift, push, pull or carry heavy demands? (>5kg) | 1 | 2 | 3 | 4 |
| 2 | Exert great force on tools or equipment? | 1 | 2 | 3 | 4 |
| 3 | Bend/ twist the wrists/hands? | 1 | 2 | 3 | 4 |
| 4 | Work in an awkward position with the wrists/hands during an extended period of time? | 1 | 2 | 3 | 4 |
| 5 | Perform short repetitive movements with wrists/hands? | 1 | 2 | 3 | 4 |
| 6 | Keep your arms up? | 1 | 2 | 3 | 4 |
| 7 | Make continuously similar movements with arms, hands or fingers every minute? | 1 | 2 | 3 | 4 |
